# Attention Deficit Hyperactivity (ADHD) and Autism Spectrum Disorder (ASD): on the Role of Alcohol and Societal Factors

**DOI:** 10.5812/ijhrba.9640

**Published:** 2013-03-12

**Authors:** Sergei V. Jargin

**Affiliations:** 1 Department of Public Health, Peoples’ Friendship University of Russia, Moscow, Russia

**Keywords:** Autistic Disorder, Hyperactivity, Alcoholism

*Dear Editor,*

ADHD predicts future substance abuse/dependence (1). In the West, the earlier shift from behavioral to neurological emphasis concerning ADHD was revised (2), while Russian literature places neurophysiologic mechanisms into the foreground. ASD cases are often marked by symptoms consistent with ADHD (3). This letter continues the paper (4), where a case of child abuse was discussed. The following risk factors of child abuse (5), were present in that case: young age of the abusing stepfather, who was 15 years older than his victim (S), maltreatment history in the abuser’s past. An ethnic factor played a role: the abuser was of Jewish descent, while S used to stress his Russian ethnicity. Having a Jewish stepfather, who even worked for some time at his school, S was often treated by the social environment as a member of the ethnic minority. It was expressed by bullying, sometimes visibly inspired by elders including some teachers and other children’s relatives. The author does not intend to say that Jewish children were generally bullied at Soviet schools. Many of them were not, because they had been better prepared, did not deny their difference, and behaved adequately. On the contrary, S behaved ambitiously, involuntarily provoking his social environment. S himself participated in bullying children from ethnic minorities, which cannot be morally justified, but is psychologically explicable in this case. His role should be classified as bully-victim, reportedly more at risk of substance use than pure bullies or victims (6). Apart from occasional participation in parties at home and drinking up to a bottle of beer with a schoolmate, S had not used alcohol till the age of 13 years, when he consumed a 750 mL bottle of a Port imitation with an older boy. During the following year, his alcohol consumption increased up to 250 mL of vodka with beer or 750 mL of fortified wine at one session. An opportunity to stay away from the abusive atmosphere at home was provided by a company of schoolmates including older boys, who inspired alcohol purchase and consumption (7). However, S did not develop physical dependence and discontinued alcohol misuse later in life. Furthermore, at the age of about five years, S presented with communication deficits, failure to develop appropriate peer relationships, motor clumsiness, and other symptoms of ASD. Some AHDH symptoms were observed as well, inattention, impulsivity and hyperactivity, the latter being more prominent in a familiar environment. Appearance of the autistic symptoms coincided with the time when the socially unskilled child became a victim of bullying. On the author’s opinion, physical abuse is an undervalued cause of ASD, some ASD children probably are battered ADHD children or initially healthy ones. A child in the atmosphere of bullying cannot permit himself some ADHD manifestations described by the collective terms impulsivity and hyperactivity. Moreover, child abuse is associated with inadequate parenting (8), therefore, heritability of ASD can be caused not only by genetic but also by social factors. Children of deviant parents may be more exposed to maltreatment, as a result acquiring deviant features themselves.
